# Real-world data in the United Kingdom: opportunities and challenges

**DOI:** 10.1186/s12916-016-0647-x

**Published:** 2016-06-24

**Authors:** Laura McDonald, Dimitra Lambrelli, Radek Wasiak, Sreeram V. Ramagopalan

**Affiliations:** Evidera, Metro Building, 6th Floor, 1 Butterwick, W6 8DL, London, UK

**Keywords:** Real-world evidence, Care.data, United Kingdom

## Abstract

Real-world data is that collected outside the constraints of controlled clinical trials and is increasingly informing decision-making in healthcare. The landscape of real-world data in the United Kingdom is set to evolve over the coming months as the government plans to build on databases currently in place by collecting patient data from all family practices and linking this information with hospital records. This initiative, called care.data, has the potential to be an invaluable resource. However, the programme has been criticized on grounds of data privacy, which has led to an extended delay in its implementation and the expectation that a large number of people will opt out. Opt-outs may introduce substantial biases to the dataset, and understanding how to account for these presents a significant challenge for researchers. For the scope and quality of real-world evidence in the United Kingdom to be realised, and for this information to be used effectively, it is essential to address this challenge.

## Background

Real-world data has been defined by the Association of the British Pharmaceutical Industry as data collected outside the controlled constraints of conventional randomised clinical trials to evaluate what is happening in routine clinical practice [1]. While clinical trials provide the ‘gold standard’ for establishing treatment efficacy, they are conducted in small populations under tightly controlled conditions and thus alone are insufficient to guide clinical practice [2]. As a result, real-world data have become important components for healthcare decision-making [3]. By operating outside the confines of clinical trials, real-world data can be used to understand treatment effectiveness as well as to provide insight into patterns of care, long-term drug safety, healthcare resource utilisation and disease epidemiology.

The routine implementation of electronic medical records, as well as technological advances that have allowed for the safe storage and sharing of data, have provided an unparalleled opportunity to gather and use real-world data to inform decision-making in healthcare. With a national health service (NHS) and general practitioners (GP) acting as gatekeepers to secondary care, the United Kingdom (UK) is uniquely positioned to collate rich data across its network of healthcare providers.

The Clinical Practice Research Datalink (CPRD) is one of the UK’s existing primary care databases, which for over two decades has extracted anonymised medical records from hundreds of GP practices and has helped to inform broad issues in patient care. The database currently has 4.4 million active patients, reflecting 6.9 % of the UK population [4]. Although working with ‘big data’ requires an understanding of how information is collected and coded, effective strategies can be developed to recode what are complex raw data for actionable scientific analysis and insight generation. Variation in disease coding (or failure to code) and differences in missing data across patients and time, as well as data not being captured at all, require effortful consideration on the part of researchers. Nevertheless, the sharing of CPRD data for research purposes has generated over 1000 peer-reviewed publications, with notable contributions including work confirming the safety of the measles, mumps and rubella vaccine [5], fracture risk associated with thiazolidinediones [6] and work demonstrating the association between body mass index and cancer risk [7].

### Care.data – opportunities and challenges

In light of the CPRD’s success and with a view to improving service commissioning across the breadth of the NHS, the government has planned to extend the reach of healthcare data collection to all GP practices in the UK, and to link this information with hospital records overseen by the Health and Social Care Information Centre (HSCIC). The initiative, called care.data, was introduced in 2013 under the tagline ‘Better Information Means Better Care’. The programme will require all GP practices in the UK to submit data, although each patient maintains the authority to opt-out and sensitive data, such as information related to sexual health, assisted conception, imprisonment or abuse, will not be extracted. As has been the case with the CPRD, patient records will be anonymised and shared with approved third parties after scientific committee approval for observational research purposes.

Care.data has the potential to be an invaluable source of real-world data in the UK. The main advantage of the larger sample size is greater statistical power and the ability to look at more determinants. This is especially important for investigating rare diseases or rarer adverse events from treatments. The database will also overcome some of the limitations of CPRD by having primary and secondary care information already linked (although some information, such as treatments given in hospital, may remain uncaptured).

Despite the potential value of the database, balancing the need for well-conducted observational research with the patient’s right to confidentially has thus far proved challenging. This was illustrated when the introduction of the care.data programme in 2013 was met by widespread criticism with concerns raised regarding the sharing of personal data without explicit consent, the distribution of sensitive data to third parties and data safeguarding. Mismanagement and poor communication from the government regarding these issues resulted in an extended delay in implementation. In September 2015, the government commissioned a review into data-sharing practices in the UK, which will go to public consultation after the EU ‘Brexit’ referendum in June 2016. When the care.data programme is eventually resurrected, a large number of opt-outs should be expected, with initial suggestions showing more than 1 million patients, or 2.2 % of the NHS’s 56 million patients already choosing to have their data removed [8]. This number of opt-outs, if occurring non-randomly, has the potential to introduce substantial biases into the extracted dataset. Understanding how to identify and account for any biases, as well as the incomplete variable coverage, presents a significant challenge for researchers using care.data [9].

Importantly, auto-enrolment with the option to opt-out offers a substantially more effective and representative method for participant recruitment than an opt-in approach. Previous work has shown that recruiting participants to an observational medical database via an opt-in strategy compared to an opt-out approach resulted in significantly lower recruitment rates and a biased sample, such that those patients included tended to be healthier on multiple indicators [10]. Equally, a Canadian stroke registry requiring explicit consent led to selection biases in key demographic and clinical characteristics known to be associated with long-term patient outcomes [11]. When creating a national resource for healthcare data, an opt-in approach to participant recruitment has the potential to leave those patients most requiring care unaccounted for.

The impact of opt-outs on medical records data, however, has not yet been well-defined. For example, in a United States birth cohort, the introduction of opt-out legislation led to significant differences in ethnicity and maternal age between families who were included and those who opted-out [12]. This suggests socio-demographic factors influence decisions to opt out, which could bias the sample. However, other work in a cohort being treated for a urinary tract infection found no differences in age, gender and one clinical characteristic (urine test) between patients choosing to opt-out and those included in the final sample [13]. These are, however, small studies (*n* < 2000) including only limited clinical indicators. Clearly, more work is needed to identify the socio-demographic and clinical characteristics of patients choosing to opt-out in order to understand how this may reduce the value of the data.

## Conclusions

Even in view of the large number of expected opt-outs, it is important to consider that surveys show most patients are supportive of medical records research [14, 15]. Indeed, Nordic countries have established national mandatory health registries with great success [16, 17]. In these regions, provision of data is seen as part of the contract for receiving free healthcare, and ethical review committees help ensure data are handled in a way that maintains patient privacy [18]. In the UK, any decision to opt-out is likely to be driven by the patient’s perception of the risks and benefits involved in sharing their data [15]. Importantly, adequately framing arguments around privacy concerns has been shown to generate more positive attitudes [19]. Therefore, appropriate information needs to be channelled to groups that may be misinformed or to those patients who may desire more information. Patients also need to be engaged, for example, by receiving information on how their data are being used [20]. However the challenge of opt-outs are met, adequately addressing the issue will be essential to ensure the scope and quality of observational research generated using care.data are not compromised, and to continue building on the successes of existing databases such as the CPRD.
